# Infectious disease emergence and global change: thinking systemically in a shrinking world

**DOI:** 10.1186/2049-9957-1-5

**Published:** 2012-10-25

**Authors:** Colin D Butler

**Affiliations:** 1 National Centre for Epidemiology and Population Health College of Medicine Biology and Environment, Australian National University, Canberra, Australia

## Abstract

**Background:**

Concern intensifying that emerging infectious diseases and global environmental changes that could generate major future human pandemics.

**Method:**

A focused literature review was undertaken, partly informed by a forthcoming report on environment, agriculture and infectious diseases of poverty, facilitated by the Special Programme for Tropical Diseases.

**Results:**

More than ten categories of infectious disease emergence exist, but none formally analyse past, current or future burden of disease. Other evidence suggests that the dominant public health concern focuses on two informal groupings. Most important is the perceived threat of newly recognised infections, especially viruses that arise or are newly discovered in developing countries that originate in species exotic to developed countries, such as non-human primates, bats and rodents. These pathogens may be transmitted by insects or bats, or via direct human contact with bushmeat. The second group is new strains of influenza arising from intensively farmed chickens or pigs, or emerging from Asian “wet markets” where several bird species have close contact. Both forms appear justified because of two great pandemics: HIV/AIDS (which appears to have originated from bushmeat hunting in Africa before emerging globally) and Spanish influenza, which killed up to 2.5% of the human population around the end of World War I. Insufficiently appreciated is the contribution of the milieu which appeared to facilitate the high disease burden in these pandemics. Additionally, excess anxiety over emerging infectious diseases diverts attention from issues of greater public health importance, especially: (i) existing (including neglected) infectious diseases and (ii) the changing milieu that is eroding the determinants of immunity and public health, caused by adverse global environmental changes, including climate change and other components of stressed life and civilisation-supporting systems.

**Conclusions:**

The focus on novel pathogens and minor forms of anti-microbial resistance in emerging disease literature is unjustified by their burden of disease, actual and potential, and diverts attention from far more important health problems and determinants. There is insufficient understanding of systemic factors that promote pandemics. Adverse global change could generate circumstances conducive to future pandemics with a high burden of disease, arising via anti-microbial and insecticidal resistance, under-nutrition, conflict, and public health breakdown.

## Multilingual abstracts

Please see Additional file 1 for translations of the abstract into the six official working languages of the United Nations.

## Background

There is widespread and legitimate concern about global environmental change (GEC), “limits to growth” and global and regional social health determinants such as worsening inequality. Allied with this growing attention to GEC is rising anxiety about emerging infectious diseases. Several aspects of GEC, including international travel, climate change, and the trade in livestock and plants have been explicitly linked to emerging infectious diseases (EIDs) in humans and other species [1-3]. This article provides a focussed summary of these two literatures and a critical assessment of their interactions.

These identified connections between two dimensions of a greater problem; GEC and EIDs matter, but the relevance of most identified factors to future infectious diseases may be dwarfed by factors whose importance is at present far less well appreciated. Indeed, it is possible that circumstances more familiar to earlier human generations could evolve, creating a fertile terrain which could enable the resurgence of currently dormant infectious diseases, of great consequence to our forebears. This context could evolve through the conjunction of worsening under-nutrition, and, in some parts of the world, impaired governance. Both factors threaten to erode the foundations of public health, an important determinant of emerging infectious diseases, recognised by pioneers in this literature [4].

The structure of this paper is conventionally arranged as background, methods, results and conclusion, however this is to an extent artificial, as no experiment was actually performed. Rather the majority of this paper presents a series of conceptual frameworks, concerning GEC and EIDs, threshold effects for physical, social and eco-social events and then potential burden of disease of EIDs.

## Method

A focussed literature review was conducted, building on substantial research conducted by the author for a forthcoming report about environment, agriculture and infectious diseases of poverty [5]. The author acted as a consultant (2008-present) for the “think tank” (called “Thematic Reference Group IV”) that undertook this report, under the stewardship of the Special Programme for Tropical Diseases Research. He also served as editor for this Report. The author’s long familiarity with the interactions between global environmental change and health [6] made the task possible.

This article extends part of the material in this report. This is based on an overview of relevant literature obtained by soliciting expert opinion, starting first with the Thematic Reference Group members. This was supplemented by a literature search using numerous relevant keywords, embracing both general and specific topics related to these topics. The main search engine used was Google Scholar because of its capacity to search diverse literatures. The search was restricted here to published journal articles and books, though for the Technical Report the literature searched included many reports. Literature in the area of global environmental change is too large to permit a systematic review, and extends far beyond that normally considered as biomedical or health-related. The author also has considerable familiarity with the EID literature. While, there was not sufficient time to conduct an exhaustive literature review into interactions between global environmental change and EIDs, it is unlikely that major relevant publications have been overlooked.

## Results

**Figure 1:**
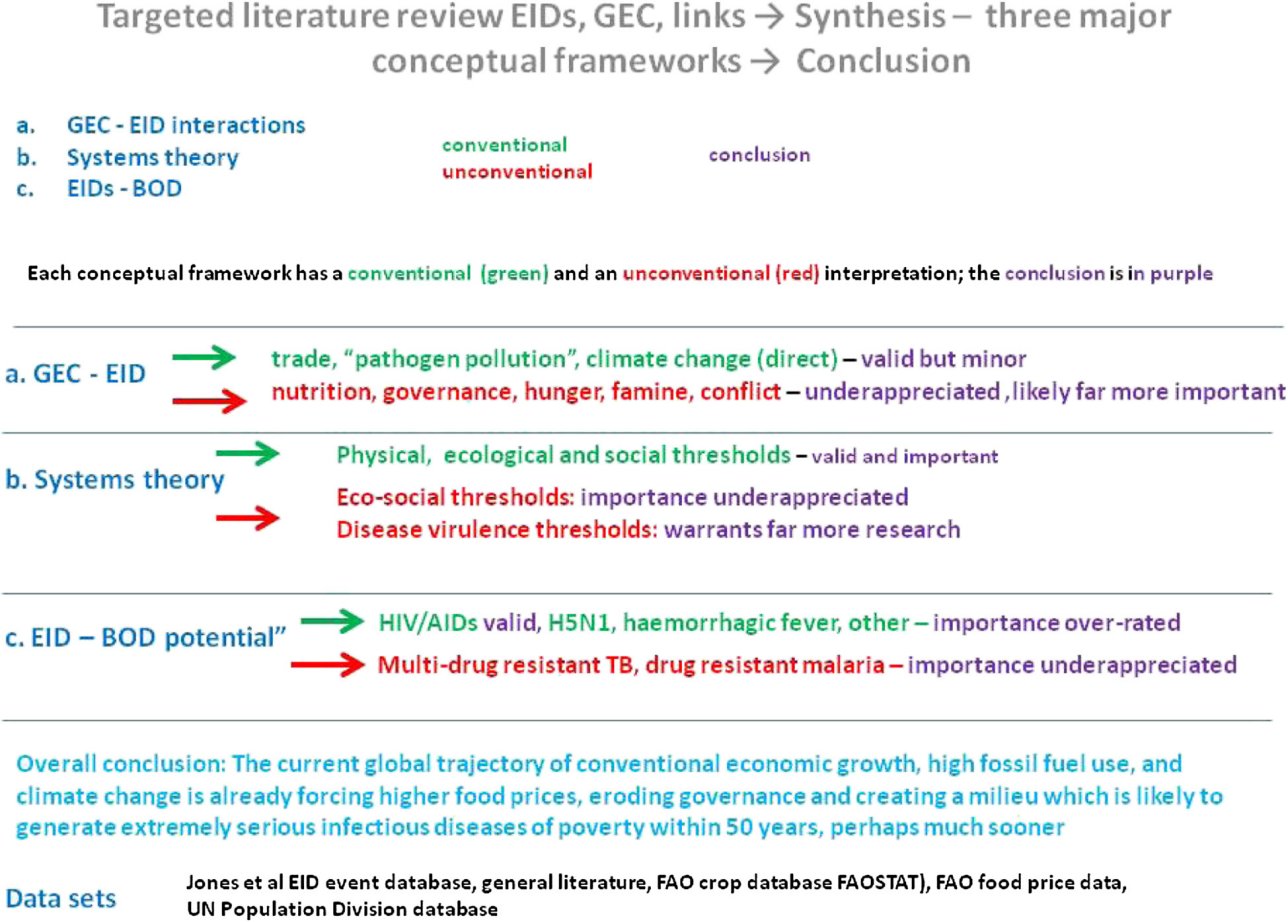
**Outline of the results section of this paper.** The paper presents three linked conceptual frameworks, leading to the overall conclusion. Several datasets are used.

**Figure 2:**
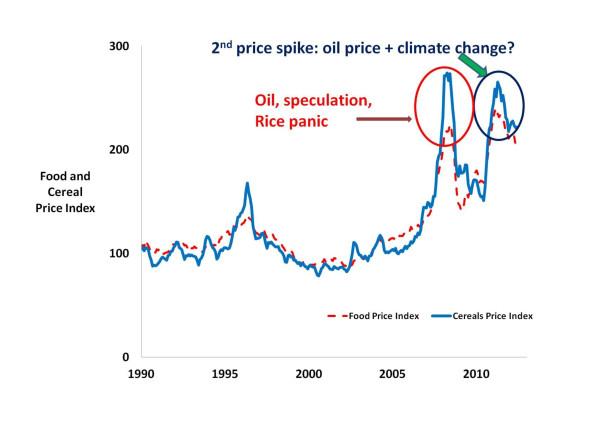
**FAO food index 1990-August 2012, Global food crises have increased substantially since 2005.** The second rise, after 2010, has probably resulted more from extreme weather events than from high energy prices, or from biofuels (raw data, FAO).

**Figure 3:**
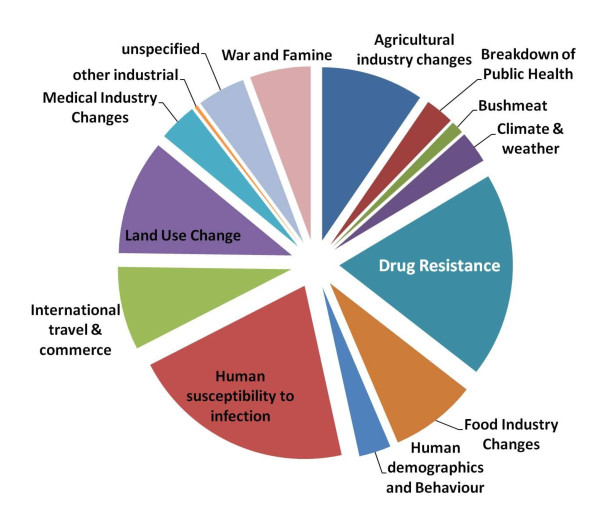
**Fourteen categories of EID events described by Jones et al. [**[7]**].**

An outline of the results section is shown in Figure 1. This section first summarises relevant aspects of global environmental change. It then discusses physical, social and “eco-social” thresholds and effects, including the relationship between current energy prices and the global recession. It then discusses global change, including how climate change has increased food prices (see Figure 2). Rising food prices impair nutrition, harm immunity and thus increase vulnerability to infectious diseases and some chronic diseases. The paper then analyses, critically, one of the most important recent analyses of EIDs, that by Jones et al. [7], focussing especially its causal classification (Figure 3). 

**Figure 4:**
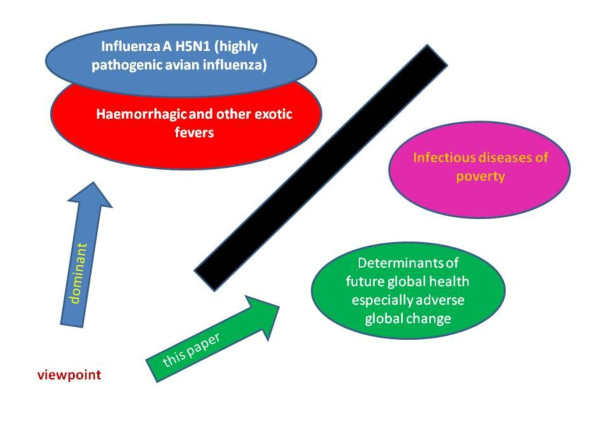
**Two perspective on EIDs and Global Change.** The dominant perspective in the EID literature is currently to look to the left of the black line in this figure, discounting the existing health consequences of infectious diseases of poverty and the future risks to EIDs due to adverse global change. In contrast this paper focuses mainly on the view to the right side of this line. However, it also argues that the public health risks of EIDs, as mostly perceived, are exaggerated.

**Figure 5:**
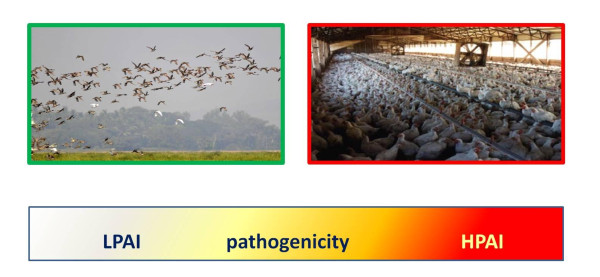
**Highly pathogenic avian influenza is more likely to evolve in the pathogenic milieu in the chicken Concentrated Animal Feeding Operation (CAFO) (right) than the wild bird flock (left), where low pathogenic forms are more likely (adapted from [**[99]**]**.

**Figure 6:**
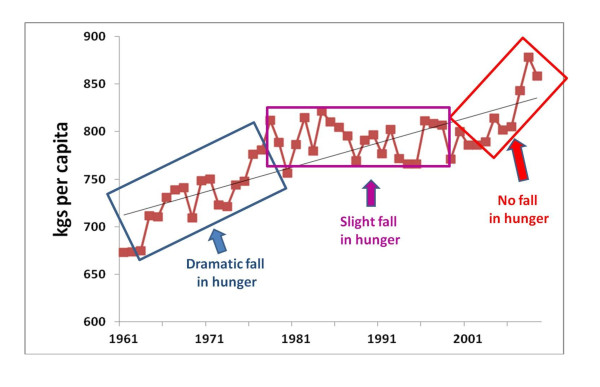
**From 1960 to about 1980 per capita production of the top 20 food crops (by tonnage) rose steeply, associated with a dramatic fall in global hunger (including as a proportion).** In the last few years per capita production of these crops has again risen, yet global hunger has worsened. Raw data FAO (FAOSTAT), UN Population Division.

**Figure 7:**
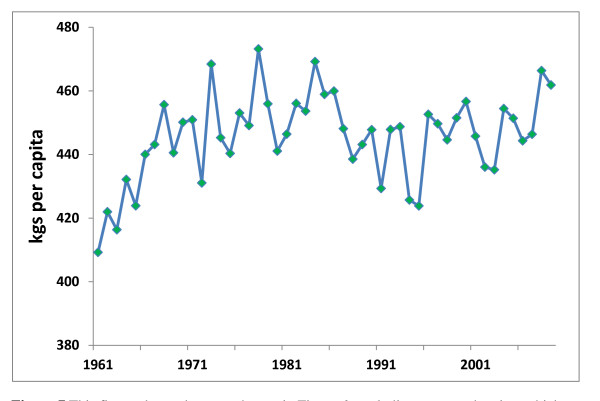
**This figure shows the same data as in Figure**6**, excluding sugar and maize, which increasingly have been used for ethanol rather than food.** The trend in per capita agricultural production is much flatter since about 1980, however the increase from 1961 to about 1980 is similar in both figures. Raw data FAO (FAOSTAT), UN population division.

**Figure 8:**
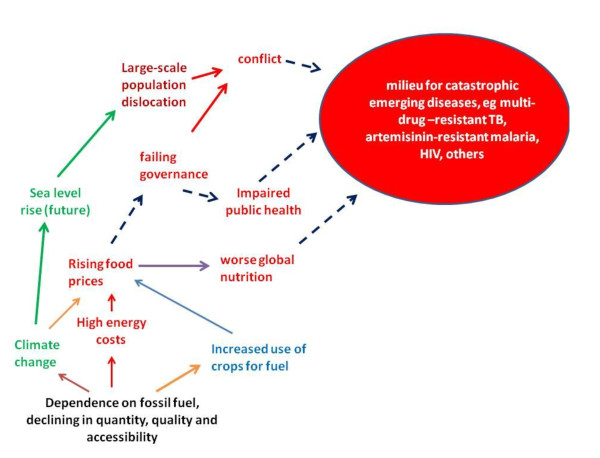
**Pathways between adverse global change and catastrophic emerging diseases.** This figure show a subset of these pathways, considered by the author to be of the greatest importance.

**Figure 9:**
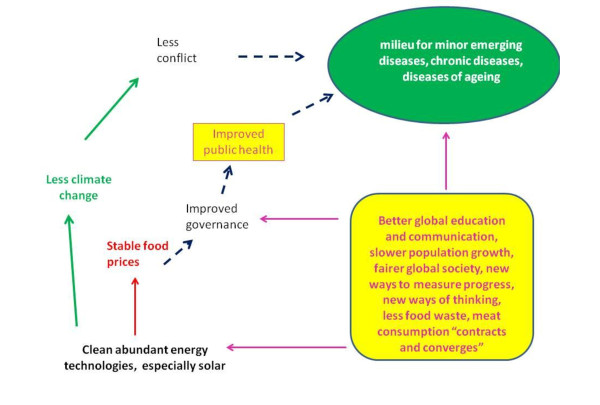
**Pathways towards a more encouraging future.** With new thinking, better leadership, less waste and new technologies, especially to produce clean and abundant energy food prices could stabilize, the rate of climate change would slow and nutrition would improve. In this milieu, progress would continue to be made to deal with existing infectious diseases of poverty, and the risk of emerging diseases with a catastrophic burden of disease would abate. The main global health problems would be of chronic diseases and diseases of ageing.

The paper then becomes more speculative, discussing the characteristics of infectious diseases that have the potential to have a high burden of disease, including a discussion of influenza, Nipah virus and HIV. It challenges the conventional focus on exotic fevers as the major risk of EIDs (Figure 4), and also discusses the ecological characteristics which may influence the evolution of highly pathogenic avian and human influenza (Figure 5). The paper then challenges more conventional wisdom – the current state of global per capita food supply (Figures 6, 7). The paper then concludes with an appeal for greater systemic thinking to reduce the potential of GEC to create a milieu for EIDs with a high burden of disease, using two scenarios, one pessimistic (Figure 8) and one optimistic (Figure 9).

In the course of this analysis, the paper uses several datasets: FAO food price data (Figure 2) the Jones et al. EID event database (Figure 3) and FAO crop database (FAOSTAT) and UN Population Division data (Figures 6, 7).

### Global environmental change

Evidence of significant human-driven planetary change is irrefutable [8,9]. Climate change receives the greatest attention, but many other planetary changes are evident, including disruptions to the nitrogen cycle, to ecosystems, biodiversity and to land use [10]. More than half of the world’s people now live in cities, and increasingly, young generations are being raised in environments with little exposure to rural ecosystems [11]. Supplies of cheap, accessible oil are declining, and a consequence is that energy prices remain persistently high, even during a deep global recession. In fact, the high energy price has been causally linked with the recession [12,13]. Energy underpins human services and the capacity of civilisation to manufacture and distribute goods. Affordable energy is essential both for economic activity as well as for cooking, lighting, transport and, in some cases, heating and cooling.

The evidence for global warming and a changing climate becomes stronger every year. The scientific consensus concerning climate change may soon rival that of heliocentricism and natural selection. However, as with earlier examples, public understanding and acceptance of these revolutions lags far behind that of science. Evidence of the reality of climate change is seen, for example, in increased global land and ocean temperatures, especially strong in the Arctic. In August 2012, Arctic Ocean ice extent fell to a new minimum. The mass balance of the Greenland ice shelf is declining at an accelerating rate [14] and is already contributing to sea level rise [15]. The intensity of rainfall is increasing [16,17], at the same time that droughts are becoming more severe [18]. These changes, with others, are making farming more difficult, for both crops and livestock [19].

Global sea level is currently rising mainly because of thermal expansion as the ocean warms, partially offset by increased water held in reservoirs [20]. Additional ocean volume and thus sea level rise due to the melting of the polar icecaps is excluded from most models and projections of future sea level. Mounting concerns now exist for the stability of the Greenland and East Antarctic Ice Sheets this century [21]. Sea level rise by 2100 could be as high as 1–2 metres compared to 2000, if the two-degree global warming guardrail is crossed [22,23].

Numerous other indicators of planetary stress exist, such as the state of ecosystems, the extent of forests, and ocean temperatures. Concepts such as “planetary boundaries” [10] the Living Planet Index [24] and the Index of Global Environmental Change [25,26] integrate these indicators and warn of unsustainable trends. Accelerating climate change is not the only trend of concern. Others include the growing scarcity of phosphate [27], worsening trophospheric ozone depletion [28], the exhaustion of fish stocks, and the decline in biodiversity [29]. These trends are already interacting with society and, unless reversed, will increasingly generate adverse health impacts [26].

### Physical, social and eco-social threshold events

There is a well-established concept of thresholds, beyond which an entire system can transform, in the natural science literature, and to a lesser extent in the social sciences. However, there is less understanding of the related concept of “eco-social” tipping points. This refers to the interaction between social and environmental events (including ecological) [30,31]. Yet, examples of all three forms of threshold are commonplace. For example, if a glass of water is knocked only slightly, it may not topple, but remain upright after some wobbling. However, if displaced beyond a threshold, the vessel will fall unless there is an intervention such as someone stabilising it.

Ecological thresholds exist on many scales. At a local level, the clearance of vegetation around a stream may lead to the local extinction of amphibious species. A sub-continental example concerns the area now known as the Sahara desert. About 5,500 years ago this region was fertile, but changes due to an interaction of climatological and land-cover factors, characterised by strong, non-linear feedbacks [32], transformed this landscape into a desert. On an even larger, planetary scale, Lenton et al. list several physical tipping points, defined as “critical thresholds at which a tiny perturbation can qualitatively alter the state or development of a system” at risk from global climate change [33]. One of these tipping points, for example, operates through a feedback whereby increased warming of the Arctic leads to the release of additional greenhouse gases, currently sequestered in the tundra, and hence to further global warming [34,35].

Social thresholds are also commonplace, and can be benign, such as laughter and applause; or, harmful, such as the onset of aggression and violence. In each of these cases, there is a point at which the “emergent” phenomenon is imminent, but not inevitable. For example, a standing ovation becomes unstoppable when a sufficient number of individuals have stood up, reaching a threshold [36]. Social pressure then forces all, or almost all of the remaining audience to also stand. The number required to reach this threshold may be lowered if the people displaying their enthusiasm are of high status and visible to the audience, such as those people who paid higher prices to be near the stage.

In addition to social or ecological tipping points, thresholds can be caused through an interaction of both. A minor example of an eco-social phenomenon on a small scale is when a crowd watching a sporting event runs for shelter during a sudden rainstorm. Individuals react not only to the rain, but the group behaviour, which may create a mood which temporarily permits, and indeed almost demands that people behave in an unusual way, that is by running. This event is not purely social; people are responding to an environmental factor; in this case an unexpected rain shower.

However, the co-mingled causal nature of many eco-social phenomena is often contested, sometimes bitterly. For example, the Rwandan genocide of 1994, [37] together with many other conflicts in Africa [38] and elsewhere are often analysed as purely social events, rather than arising due to an interaction between ecological events (especially resource scarcity, most often of land) and social factors [39,40]. The tension between Israel and its Arab neighbours is very rarely analysed to have an environmental dimension, especially by Israelis [41]. Instead, the tensions are generally considered to have a purely social explanation, based on culture and religion.

Migration, including the seeking of political asylum, is also frequently characterised as having a purely social and “economic” causation [42]. Yet, economic factors are associated with elements that are both material (e.g. food, shelter) and social (e.g., freedom of association and speech, psychological security).

A current, large-scale, eco-social event may be the contribution of sustained high energy prices to the global financial crisis [13]. The prices of oil and food have risen sharply since 2008. Fatah Birol, chief economist of the International Energy Agency agrees with Sir David King, the recent U.K. chief scientist, that we are already in the era of “peak oil”, though not yet peak fossil fuel, due to the temporary glut of non-conventional oil and gas such as tar sands, deep water oil, Arctic oil, and shale and coal-seam gas [43]. The abundant scale of reserves of coal have also been questioned [44]. In any case, without extraordinary technological breakthroughs, currently unforeseeable, real energy prices seem unlikely to return to the levels seen through most of the 20^th^ century [45].

### Global change, food prices and infectious disease vulnerability

After more than a century of decline [46], the global price of wheat and other cereals rose sharply in 2008 (see Figure 2). This was accompanied by rises in the cost of numerous other foods [47], sufficient to tip many additional people into hunger and precipitate food riots in several nations [48]. The main reason for the rise in food price at that time was that oil reached over $140 a barrel, during a frenzy of commodity speculation which included food and fertilizer [47]. At the World Health Assembly that year, the rising food price was named as one of the three major emerging global health threats, the others being climate change and pandemic flu [49]. Then, in 2009, the price of both energy, fertilizer and food fell, as the global financial crisis tipped hundreds of millions of people into poverty and hunger [50].

Yet, in December 2010, the global food price exceeded the 2008 record, and the cereal price almost did too (see Figure 2). However, the price of oil was less than US$100/barrel at that time. This second food-price spike has, as yet, generated little published analysis. Proximal causes are likely to include comparatively high energy costs and the incremental increase in the fraction of the global food supply diverted to biofuels [51]. However, an increasing number of researchers have argued that the 2010 heat wave in Russia and the Ukraine, and the floods in Pakistan, were worsened by climate change [52-54]. The year 2011 also saw abnormal heat and drought in large parts of the USA, along with severe flooding again in Pakistan and Australia, and also in Southeast Asia. Severe drought in the Horn of Africa, combined with inadequate governance, contributed to extensive famine in Somalia. That drought has been linked to climate change [55,56]. In July 2012, the global food price increased again, due largely to the drought and record high temperatures of the 2012 USA summer, which substantially reduced its corn and soy crops. That event is too recent to be attributed by climatologists to the level of atmospheric greenhouse gases, but it is consistent with these other events [54].

When food prices rise, poor populations preserve calorie intake at the expense of nutrients, increasing their vulnerability to infectious diseases, especially by reducing their immunity [57,58]. They are also likely to reduce discretionary spending, including health care. This also increases vulnerability to infectious diseases. Food price rises also increase the rate of stunting among vulnerable populations, especially, in some cultures, among female infants and children. Prolonged under-nutrition in childhood, whether from malabsorption, chronic parasitic infection, inadequate food intake or, more commonly, a combination of all three factors increases the likelihood of chronic diseases emerging at an earlier stage in later life, including diabetes and other conditions that predispose to infectious diseases, whether emerging, re-emerging or dormant (like plague).

### Emerging infectious diseases

Allied with the growing concern about GEC is rising anxiety about emerging infectious diseases. From World War II until the 1970s, improvements in living conditions, knowledge, insecticides and medical technology, especially vaccines and antibiotics, enabled an unprecedented global retreat of important infectious diseases. Smallpox was eradicated, apart from its containment in two secure laboratories [59]. This progress was particularly strong in developed countries, where infant or child mortality became a rarity. But, much progress was also made in Asia, especially with malaria. Widespread availability of effective and cheap antibiotics lowered the burden of respiratory infections, and oral rehydration solution provided affordable treatment for diarrhoeal diseases. Health for All appeared, briefly, to seem achievable [60,61].

At least two U.S. Surgeon Generals made premature, optimistic, but rich country biased forecasts of the demise of infectious diseases [62]. In 1969, Surgeon General William Stewart announced the time had come to ‘close the book on infectious diseases’. In 1979 Julius B. Richmond announced that infectious diseases were the ‘predecessors’ of the degenerative diseases that succeed and replace them. According to Snowden, the course of nature, in Richmond’s view, was “simple, uni-directional, and benign” [62].

But, a later Surgeon General, Everett Koop, who served two terms under the conservative US President Ronald Reagan, helped redeem this over-optimistic bias by recognising, comparatively early, the risk that HIV/AIDS posed to USA and global public health. In 1988, Koop oversaw production of the brochure *Understanding AIDS* and mailing to the nation’s 107 million households [62].

The salutary experience of HIV/AIDS helped to energise concern over infectious diseases in developed countries, bringing recognition that neither wealth nor distance could guarantee complete safety. This led to several important publications in the USA in the early 1990s on the topic of what we now call emerging infectious diseases, including by the US National Academy of Science, the US Centers for Disease Control and Prevention, and Stephen Morse’s book *Emerging Viruses*[63]. Soon after, the US Centers for Disease Control and Prevention established the journal *Emerging Infectious Diseases*, which first appeared in 1995 [64].

In the first issue of this journal, Morse defined emerging infectious diseases as “infections that have newly appeared in a population or have existed but are rapidly increasing in incidence or geographic range.” Today, this journal is ranked fifth of 70 infectious disease journals worldwide and receives “millions of hits”. The term “emerging infectious diseases” on Google currently retrieves 2.3 million records. There can be no doubt that EIDs elicit intense interest.

### The Jones et al. database of 335 EID “events”

One of the most widely cited papers on EIDs was published in *Nature* in 2008 [7]. According to Google Scholar, it has been cited 794 times to date (September 13, 2012). It analyses a database of 335 EID events detected between 1940 and 2004, classed into 14 separate causal categories. Two leading causes represent forty percent (134/335) of the total. These are “human susceptibility to infection” and antimicrobial resistance (AMR) (19.1%) (see Figure 3). Remarkably, I am currently unaware of any published work that is critical of this ground-breaking paper; and a search on Google scholar using terms such as “Jones Nature emerging diseases criticism”, “Jones Nature emerging diseases critique” and “emerging infectious diseases critique” reveals nothing relevant.

Despite its prominence, this paper has several flaws. Errors and problematic statements begin with the meaning of an EID event, defined as “an infectious disease emerging in human populations for the first time”. But, their list includes 64 examples of anti-microbial resistance, often of the same organism (e.g., seven forms of drug-resistant *Haemophilus influenza)*. Diseases caused by various forms of the same organism are not qualitatively distinct; a further issue is that many diseases, from pneumonia to cystitis, have multiple microbiological causes. The database Jones *et al.* uses what might be more accurately called a collection of emerging diseases and pathogens with newly-identified forms of anti-microbial resistance (AMR). But, to claim that only 64 forms of drug resistance have been identified since 1940 is clearly a gross underestimate, probably by orders of magnitude.

### Drug resistance and burden of disease

Many of the AMR conditions are highly specific. For example, *Acinetobacter baumannii* accounts for four events, as strains have been found resistant to three antibiotics (gentamycin, imipenem, and polymixin) with the fourth “event” being a multiple drug resistant form. Carbapenem resistant *A baumannii* also exists [65], and this would thus be a fifth EID event involving this species alone. But, while this bacterium is problematic for severely immuno-compromised individuals, such as those in intensive care units, its current (and past) burden of disease is low, or even trivial at a global scale (see Table 1). Its *potential* burden of disease also appears to be modest. 

**Table 1:** EID examples from the Jones et al. data base

**Pathogen**	**Jones et al. classification**	**Suggested classification**
Chikungunya virus 1952 (Tanzania)	Human demographics & behaviour#	Ascertainment
Crimean-Congo Hemorrhagic Fever 1944 virus (Soviet Union)	War & famine	Ascertainment
Dengue 1954 (Philippines)	War & famine#	Ascertainment or travel
Ebola virus (1976 Sudan)	Bushmeat*	Ascertainment
Guama 1954 (Brazil)	Land use changes	Ascertainment?
Guanarito 1989 (Venezuela)	Land use changes	Ascertainment?
Hantaan 1941 virus (China)	Land use changes	Ascertainment?
Hendra virus 1994 (Australia)”	International travel and commerce#	Land use change
Hepatitis C 1975 (USA)	Human susceptibility to infection#	Ascertainment
HIV/AIDS 1959 (Congo)	Bushmeat*	Ascertainment
Lassa fever 1969 (Nigeria)	International travel and commerce#	Ascertainment
Murray Valley Encephalitis 1950 (Australia)	Climate and weather#	Ascertainment?
Nipah 1998 (Malaysia)	Agricultural industry changes *	Novel
Nipah 2001 (South Asia)	not listed	Ascertainment
Plasmodium vivax 1964 (India)	Breakdown of public health measures	Insecticide resistance

This table includes one EID (Nipah in South Asia) that was not included by Jones et al. Several classifications appear accurate and useful (*), others are wrong (#). Many were probably ascertained in this period (1940–2004).

The potential disease burden of AMR varies with the characteristics of the microbe and the nature of the antimicrobial resistance. For example, multi-drug resistant *M tuberculosis*, or artemisian resistant malaria have enormous disease potential, but many other forms of drug resistance are neither important from a public health perspective nor informative from a biological viewpoint.

Others in this AMR category have an obviously high burden of disease such as HIV-1 (listed four times, once due to “bushmeat” and three times due to different forms of drug resistance) and multiple drug resistant *Mycobacterium tuberculosis* (listed three times, though only twice in the AMR category). Another potentially catastrophic form of drug resistance is of artemisin resistant *Plasmodium falciparum*, the causative agent of the most deadly form of malaria. Treatment with sub-therapeutic doses of artemisinin monotherapies for over 30 years, together with substandard (and even fraudulent) medication, have probably been the main driving forces leading to this emergence in the Thai-Cambodian border [66,67].

Many other anomalies and problems exist. Another example is that the database lists six forms of resistant *P falciparum* and three “events” relating to *P vivax.* Confusingly, while AMR involves eight of these malaria-related events, two are grouped in other categories, as land use change and war and famine. The ninth, a resurgence of *P vivax* in India, is probably due to insecticide resistance, but there is no category for this in this database, though some earlier classifications of EIDs explicitly included insecticide resistance [68]. It is instead classed as “breakdown of public health services” (see Table 1).

### Misclassification

Other conceptual errors, including numerous misclassification errors (see Table 1) reduce the analytical value of this paper. The classification of some diseases also suggests a bias towards a developed country perspective. For example, Lassa Fever (first identified in 1969, after an outbreak at a hospital in Nigeria) is attributed to “international travel and commerce”. While it is true that Lassa Fever may be disseminated to other nations, rich and poor, through air travel, this was not so for the 1969 EID event. In addition, the vast majority of Lassa Fever cases remain in West Africa, where the animal reservoir exists.

Several other newly identified EIDs are attributed to a variety of causes, but are quite likely to be newly identified (ascertained) rather than genuinely new, such as new variant Creutzfeld Jakob Disease. Another pathogen that probably is genuinely novel in human populations is Hendra virus. However, this is misclassified as due to “international travel and commerce”.

The database used in this paper includes several infections that have almost certainly been present in humans for many years, if not millennia, and which do not appear to have increased their historic range or incidence. Examples in this group include African haemorrhagic fevers such as Lassa Fever and Ebola, and the mosquito-borne Murray Valley Encephalitis (Australian encephalitis). While these diseases were first recognised as pathologically and epidemiologically discreet entities since 1940, it is unlikely that they have crossed into human populations only since then. They are spread by close contact, but their reproductive rate (R_0_) is less than 1; while clusters may initially occur, sustained spread is limited by barrier nursing or simply by fear, which inhibits close contact as soon as the contagious nature of the disease is realised. Their burden of disease remains comparatively low.

Other categories of EIDs described in this article are pathogens that “have recently entered human populations for the first time”, such as HIV-1, and “pathogens that have probably been present in humans historically, but which have recently increased in incidence” (for example, Lyme disease). The database used in this paper also includes several infections that have almost certainly been present in humans for many years, if not millennia, and which do not appear to have increased their historic range or incidence.

Haemorrhagic fevers have characteristics warranting of a fearsome individual reputation, but do not justify extreme public health concern. After their manifestation in people, they can be spread by close contact, but their reproductive rate is not sustained above one. Initially, disease clusters may occur, but sustained spread is limited by either barrier nursing or by fear, both mechanisms generally inhibiting sufficiently close contact for the carer or family to themselves contract the infection. These human responses appear soon after the spectacularly contagious and potentially fatal nature of the disease is realised. Thus, the burden of disease remains comparatively low and is likely to remain low.

In contrast, some diseases, including influenza, HIV and tuberculosis do justify high public health concern, because in each case infected people can be contagious without appearing so sick as to be shunned by others or to require barrier nursing. This “stealth phase” increases the chance of the pathogen spreading to others.

### Lay concerns over EIDs, Nipah virus and its sustained infection

The concern about EIDs in the scientific literature is amplified by the lay press, especially in developed countries. Anxiety is particularly high about Influenza A H5N1 (avian influenza), concern for which has been suggested and contributed to by conflicting interests, particularly from the pharmaceutical industry [69]. However, anxiety extends far beyond influenza [70]. For example, a recent article in the *New York Times* on EIDs quoted one infectious disease specialist as stating: “Nipah is spilling over, and we are observing these small clusters of cases — and it’s a matter of time that the right strain will come along and efficiently spread among people” [71].

Nathan Wolfe, author of *The Viral Storm*[72] was named by *TIME* magazine as one of the 100 most influential people in the world for 2011, for his global viral forecasting work. His book focuses on the risks of viral epidemics, especially emerging from contact with exotic wildlife, such as bushmeat. This award supports the lay perception that these “emerging viruses” are vitally important; in fact, from a public health perspective, they are unlikely to be. A focus on such exotic conditions has an opportunity cost (see Figure 4). It diverts attention and funding from the control of other infectious diseases of far greater public importance, especially neglected tropical diseases [73,74].

An unprecedented outbreak of Nipah virus did occur in Malaysia and Singapore in 1998 and 1999, with over 100 human deaths [75]. Its epidemiology is now thought to be well understood as being due to a temporary interaction (reversed by public health intervention) between bats, mangoes, farmed pigs and their farmers [76]. However, large populations of bats, mangoes and pigs had co-existed and interacted for about two decades in Malaysia prior to the identified outbreak [76]. While an earlier hypothesis linking the outbreak with the strong El Niño-associated fire season and altered bat migration of 1997–98 [77] has now been discredited [76], it is still possible that increased stress in bats (caused by deforestation) has weakened bat immunity [78], allowing more viral spill-over to pigs in the late 1990s than previously.

The pigs developed a previously unknown neurological and respiratory illness which was then transmitted to farmers [79]. Most importantly, public health intervention lowered the chance of its recurrence, and Nipah in humans has not since been detected in that part of the world. But, this outbreak of Nipah is a classic case study of an emerging disease, and probably close to what the public would recognise as an EID event.

Following this outbreak, isolated cases and occasional clusters of Nipah virus have been identified in parts of Bangladesh and the nearby Indian state of West Bengal [80]. The epidemiology in South Asia is different from that in Malaysia. Neither pigs nor any other intermediate animal are involved; instead, it is believed that patients acquire the virus from direct, accidental contact with bat secretions, such as by children playing under trees in which bats are roosting, or by contact with bat saliva from date palm sap collected in trees, but contaminated by bats who also like its taste. While some cases of Nipah in Bangladesh remain unexplained, most occur in carers of people who fall ill, or of family members who have close contact with the dying patient, as a form of respect. In this regard, its mechanism of transmission is like that of haemorrhagic fevers. This form of transmission was not reported in South East Asia, most probably because of a higher standard of medical care, and perhaps cultural differences which reduced close contact with family members. But, while sustained transmission of Nipah for five generations in South Asia has been reported [80], claims of imminent, indefinite “efficient” transmission of Nipah should be treated with reserve.

Nipah virus in South Asia is unlikely to be a new disease, but a newly *ascertained* infection, present for generations, perhaps since the inception of date palm sap collection. In resource-scarce settings such as rural Bangladesh, many illnesses lack a specific laboratory-supported identification. Until 1998, no laboratory in South Asia could have identified Nipah virus, even if they had received a specimen for investigation.

Lay concerns about EIDs are understandable, not only because of the recent experiences of HIV, SARS and the more distant cultural and historical memories of the Spanish Flu and the Black Death. The panic over the outbreak of plague in Surat, India in 1994, in which thousands of people fled the city, suggests a deep culturally embedded fear of epidemic. Like disgust, this aversion and fear could even be biological [81]. The arising of a new EID, with a high burden of disease cannot be fully excluded. However, a major argument of this paper is that the nature of such an event can be better understood than is currently the case, and also that much of the current concern is at the cost of other issues which are of more importance.

### Mechanisms of sustained infection

The mechanisms which lead to the development of sustained transmission of infection in a new species are unclear, but not entirely unknown. For example, recent experiments with H5N1 influenza appear to have successfully facilitated the evolution of a strain which can be indefinitely transmitted between non-immune ferrets [82]. This was done by a well-established virological technique known as *serial passage*.

There is also evidence that Nipah virus transmission among pigs was more likely to develop in larger rather than small piggeries [83]. In this case, the dense pig population may have reached a threshold of size, creating a natural laboratory permitting serial passage, circumstances denied in a small piggery where the disease would have burned itself out before evolving the characteristics to generate and enable sustained transmission among swine. Alternatively, the virus may not have been altered by the more densely populated setting in the larger piggery, but the larger population simply sustained a higher number of porcine infections, eventually leading to the inadvertent infection of humans. If this hypothesis is true then the R_0_ for Nipah among pigs would be greater than one.

Unlike the common cold or influenza, Nipah has no stealth phase – that is, it is not contagious while people are asymptomatic. It is also not easily spread by respiratory droplets, though respiratory transmission indeed did occur in Malaysia piggeries, possibly evolving due to serial passage in pigs. There are also reports of respiratory transmission in South Asia, and suggestions that this form is more contagious than encephalitis [80].

If the first hypothesis (that serial passage can alter the characteristics of the virus) is true, then conceivably techniques of bio-warfare could be used to serially passage Nipah among a sufficient number of humans, enabling a form to evolve with high lethality and an R_0_ greater than unity. Normally, however, serial passage leads to pathogenic attenuation, so that even if a higher R_0_ should evolve, it would likely have a lower lethality.

A mechanism, however, with an inadvertent similarity to this thought experiment has been postulated to explain the evolution of Spanish influenza among crowded, under-nourished troops at the close of World War I (WWI). Oxford and colleagues argue that the unique environment of that dreadful conflict may have created conditions in which the normal evolutionary tendency to attenuation was reversed. They theorise that so many humans were available to be infected, being in such close proximity and immunologically weakened, that pathogens with increased infectivity (in this case H1N1 influenza) may have had a competitive advantage over slower-acting rivals. Their proposal is highly speculative and has so far received little support. However the fact remains that both the milieu of WWI and Spanish flu were both extremely unusual. While these two events may be coincidental, they may also be related in ways which are not understood, including by Oxford et al. This is discussed in more detail, below.

### Two main categories of EID concern

Although the Jones database gives no indication of the burden of disease of EIDs, and though very little other work exists on this topic, other evidence indirectly suggests two main categories of EID concern. Most important is the perceived threat of novel or newly recognised infections, especially viruses that arise or are newly discovered in developing countries and that originate in species exotic to developed countries, such as non-human primates, bats and rodents. These pathogens may be transmitted by insects or bats, or via direct contact with bushmeat, probably associated with its butchering. They include haemorrhagic and other forms of fever, whether from rodents (e.g. Lassa), or bats via primates (e.g. Ebola, Marburg), [85] pigs (e.g. Nipah), horses (e.g. Hendra) [86] or civet cats (possibly SARS) [87]. Perhaps of significance, there is little concern about the potential for spread of the bat (and canine)-transmitted virus, rabies [88], even though it has a fatality rate (unless treated by post-exposure vaccination) of 100%. This is probably because of the much longer recognition of this condition, and greater epidemiological confidence of its limited potential to develop human to human forms of transmission.

The second category of greatest concern is of new strains of influenza A, arising either from intensively farmed birds, especially chickens with Highly Pathogenic Avian Influenza (HPAI) [49,89] or pigs, or emerging from Asian “wet markets” where several species of bird have close contact [90].

The apprehension about a future human pandemic of H5N1 is not just of an ordinary influenza pandemic such as those occurring every few decades for several centuries [91], most recently in 1968 [92], but something rivalling the 1918–20 pandemic commonly called Spanish flu [83], and with a potential financial cost of over two trillion dollars [93].

At first impression, both categories seem to warrant legitimate concern due to two great pandemics of the last century: HIV/AIDS (which originated in bushmeat in Africa before emerging globally) [94] and Spanish influenza, which killed up to 2.5% of the human population at the close and immediately following World War I [84,89]. Although many influenza deaths were from secondary bacterial pneumonia, treatable if antibiotics had been available, it was by far the most devastating flu epidemic known to date.

A few sceptics, however, have cast doubt on the hypothesis that HPAI could generate a new version of the Spanish flu. Prominent among these sceptics is the leading evolutionary biologist Paul Ewald who has suggested that if H5N1 does acquire sustained human to human transmission, it may reduce its capacity to kill individual humans [95], as in happens in most forms of serial passage. More recently, Palese and Wang [96] have argued that the case fatality rate among humans infected with HPAI may be orders of magnitude lower than suspected, if based on surveys of people exposed to lower viral titres.

The burden of disease in the case of Spanish flu may, however, be more because of the unique context in which it evolved than its intrinsic danger, as discussed above and below. The milieu for HIV in sub-Saharan Africa was also especially conducive to the epidemic’s establishment [97-99]. These factors included a high frequency of concurrent sexual partnerships and limited male circumcision. Both conditions helped to “fuel a chain reaction of rapid transmission from one highly infectious, newly-infected, person to another” [99].

### The milieu of Spanish flu

Oxford *et al*. have suggested that the extraordinarily high lethality of the WWI flu (commonly called the Spanish flu because Spain was not involved in that great struggle – the Spanish called it the French flu [83]) may have evolved because of the appalling conditions of the Western Front in the Great War [84]. The virus may have reached the Front around 1916 (perhaps from Kansas), and then maximised its human killing power before being seeded globally, including in densely crowded troop ships that may have allowed the virus to maintain high lethality and human transmissability. There were also opportunities for viral mixing in the army camps between humans, chickens, ducks and pigs. But, perhaps the most fundamental difference between this setting and any time either before or since was the densely populated number of humans – many with poor nutrition, some with co-existent infection such as typhoid and almost all with psychic stress – in proximity to the flu virus. Some also had been exposed to lung- and eye-damaging toxins, including mustard gas and phosgene) [100].

### The milieu for avian influenza

There is increasing appreciation that different ecological settings with different milieus favour either low or highly pathogenic avian influenza (HPAI) [101]. HPAI seems invariably more likely to evolve in dense populations of young birds similarly lacking in immunity than in wild flocks, which have a greater variety of nutrition, age, immune status, space and freedom (see Figure 5). However, among wild fowl such as geese and ducks, avian influenza is primarily transmitted oral-faecally than by the respiratory route [101]. Nevertheless, HPAI seems less common in small than in large flocks of chickens, for which primary transmission is respiratory.The viral trade-off hypothesis, developed by Ewald and others [102], speculates that evolutionary forces in most ecological situations are likely to drive pathogens, which trade off rapid host lethality in exchange for relatively prolonged host longevity. Over time, this is likely to favour greater opportunities for pathogenic reproduction. Extremely rapid host mortality is likely to result in a self-limiting epidemic, especially for pathogens which are not infectious until hosts are symptomatic. This is not the case for influenza, which as mentioned can be effectively transmitted when symptoms are either minor or have not appeared. Stealth infections like HIV/AIDS may have years or even decades to propagate to a new host before the original one dies. Syphilis is also thought to have rapidly evolved into a more benign form on introduction to European populations, allowing enhanced chances for propagation undetected by its next victim [103].

In industrial farms, however, sometimes called Concentrated Animal Feeding Operations (CAFOs), evolutionary factors may alter this calculus [83,104]. If there is a very large number of immunologically naive hosts (e.g., birds) that can be infected, and which are in close proximity, then a pathogen that causes rapid infection may be favoured, even if it causes death – as long as there are sufficient other vulnerable hosts which can, in turn, be infected before the original host dies. That is, the evolutionary penalty for pathogens that kill rapidly is lesser, even if they kill the host. Indeed, slower acting pathogens may be at a disadvantage, as the host may die before it can acquire an infection with a more extended life cycle.

If this theory is correct, then modern human conditions very rarely create the right circumstances for breeding a devastating human flu strain on the scale of Spanish flu. But this could change in future.

### GEC and future EIDs in a future environment of more extreme global health determinants

Global public health appears to be failing or at risk in several dimensions. First, there has been a persistent, poorly recognised failure to improve global human nutritional status since the mid-1990s [105,106]. There is much confused analysis of this topic, including from Josette Sheeran, head of the World Food Programme. In 2011, she wrote “Never before has food been so abundant. Global agriculture produces an estimated 17% more calories per person than it did 30 years ago, despite a population increase of more than 50%” [107]. This is a poorly informed statement, principally because the data upon which she is basing this claim are not transparently adjusted for either animal feed or biofuels. Already, almost 40% of maize grown in the USA is used to make ethanol, though a small amount is recovered as distiller’s grain and is used to supplement animal feed [108]. A growing number and quantity of other important crops including sugar cane, palm oil and cassava are not grown exclusively for human ingestion, but instead also to fuel vehicles [109]. After exclusion of sugar and maize (major bio-fuel sources), global *per capita* crop supplies are revealed as either not rising at all, or rising only very slightly (see Figures 6, 7).

GEC thus threatens to further worsen global food security and in turn act as a "risk multiplier" to stimulate millions of new refugees and harm governance in vulnerable nations. Living standards in many parts of the world already seem to be in decline, not only in low-income countries with rapidly increasing populations such as Nigeria [110] and Yemen [111], but also in many developed countries. Economic crises, recessions and economic scarcity increasingly affect large parts of Europe and the USA. The former chief scientist of the United Kingdom (UK), Sir David King, has recently attributed this decline, in part, to the rising cost of energy [13]. His co-author and he point out that Europe and the USA each spend about one billion dollars a day on importing energy. This represents a significant, sustained, and increasing diversion of purchasing power from the European and American economies.

The second risk to global public health arises from increased per person resource scarcity, on a global scale. This has been forecast by many visionaries, including some health workers [112-115]. The ecological and environmental foundations of civilization thus appear to be at risk. The emerging scarcity of raw materials extends far beyond energy. Scarcity of phosphate [27] – essential for fertiliser - and rare earths – needed for the New Industrial Revolution that optimists anticipate [116] are of particular concern. This combination is consistent with the predictions made by Meadows et al., in their report on Limits to Growth, for the Club of Rome [117]. Like Paul Ehrlich (the biologist who had earlier published *The Population Bomb*), the Club of Rome was ridiculed for several decades, since about 1980, but more recent analyses reaffirm that business-as-usual may well cause the collapse of civilization by about the middle of this century [118,119]. In turn, rising scarcity threatens to interact with conflict, the ancient response of humans and many other species to scarcity [120,121].

It could be that the combination of high birth rates [122], especially in low-income settings, resilient poverty, resilient inequality, and ever-worsening crowding could interact with climate and other forms of adverse global environmental change to breed one or more mega-pandemics. Growing eco-social stress, culminating in large-scale conflict, could generate civilisation failure, perhaps initially in niches which then coalesce or spread, most plausibly in pockets of sub-Saharan Africa, South Asia, or North Korea. These areas already contain many densely crowded human populations, with substantial under-nourishment and impaired immunity. A key ingredient of this would be a scarcity of health care, and perhaps even the non-availability of effective antibiotics together with an unlearning of basic quarantine and isolation (see Figure 8). But, in such a world, a mega-pandemic would not be the world’s only problem.

### Systems thinking and the pathogenic milieu

Systems thinking (see Table 2) can also be applied to pandemic flu, especially of its ecological and immunological environment. The pioneering microbiologist, later planetary ecologist, René Dubos believed that his first wife succumbed to tuberculosis in part consequent to the anguish she experienced when thinking of her family in occupied France; her internal condition allowed the dormant, sequestered tuberculosis bacillus to re-activate. In so doing, Dubos was retracing some of the ideas of his countryman, Claude Bernard, a leading French physician and scientist in the 19^th^ century, who contributed both to the development of the scientific method [123] and the internal milieu or terrain, later developed by Walter Cannon as homeostasis [124]. 

**Table 2:** Concepts of systems thinking

**Systems component (synonym)**	**Example**
Threshold event (emergence, tipping point, surprise, shock)	Birth, death, laughter, standing ovation, riot, violence, genocide, disease appearing in a new species or context
Positive feedback (amplifier)	Multi-organ failure, release of greenhouse gases from Arctic triggered by warming, leading to more warming; melting of icereducing albedo (reflectivity); also stimulating more warming
Negative feedback (dampener)	Physiological means to maintain homeostasis (eg appetite, thirst,renal function, sweating, shivering); social means such as fair distribution
Self-organisation	Ant nest, predator–prey ecological system, embryogenesis, many complex social systems, eg totalitarianism, democracies, cults,

This table shows examples from health, physiology, ecology and society.

It is claimed, though there is some controversy, that near the end of his life, Louis Pasteur said: “Bernard was right – it is the milieu, or terrain, not the microbe that really matters.” But, in reality, both terrain and pathogen matter. A person with a severely stressed internal milieu cannot die from tuberculosis without coming into contact with the bacillus. And, some pathogens have greater intrinsic “killing power” than others.

### Conclusion systems thinking, solutions, and “One Health One World”

While critical of some aspects of the EID literature, the main finding of this paper is that the evolution of GEC could create an adverse milieu, harming nutrition globally, and in some parts of the world, impaired governance. Both factors threaten to erode the foundations of public health, an important determinant of EIDs, recognised by pioneers in this literature [4].

Interventions and changes necessary to improve sustainable population health include improved eco-social health determinants and greater equity, new measurements of progress [125] and “co-benefits” that can help both health and the global environment [126]. Examples of these include “contraction and convergence” [127], in which affluent societies waste less, use more active transport and eat less meat [128], while poor populations increase their literacy, consumption of animal products and improved housing. In addition, a vast amount of food (including animal products) is wasted before harvest and more is lost due to poor storage. A large amount of edible food is deliberately thrown out of supermarkets, in order to promote and to preserve images of an abundance of fresh food [129].

Civilisation is operating on an outdated, archaic economic system, which evolved with and was far better adapted for a world from several decades ago, before the planet became so populated. This dominant economic system both denies limits to growth and incorrectly measures costs – especially many forms of natural capital depletion – as increases in wealth [125,130]. While perhaps hundreds of millions of people are aware of components of Earth system change, such as ecosystem depletion, climate change, over-crowded slums and poverty, very few people are integrating and communicating these risks. There are additional barriers, such as disciplinary separation and a scarcity of journals that publish multidisciplinary work.

A Green Economy, also known as “GDP plus”, is urgently needed, but this call has repeatedly been made by visionaries since at least John Stuart Mill, in 1848 [131], but not heeded. Civilisation is also marked by immense social inequality, which breeds civil stress, terrorism, [132] fascism, and war.

In this article, I have argued that health workers need to think more broadly and more ecologically. I have described several ways in which global eco-social population health determinants may worsen. But, such deterioration is not inevitable. Key ingredients for solutions that are urgently required are better leadership and technology, especially energy sources that do not negatively impact the climate, most probably from a rapidly evolving range of renewable energy technologies [133,134]. If sufficient renewable energy were available, it could be used to desalinate water and also pump Africa’s abundant ground water, improving food security in Africa [135]. New agricultural techniques using sea water for soil cooling in hot areas and other forms of bio-saline crop growth are also under development [136], though, as with many commercial ventures, the full potential remains unknown, due to commercial confidence and the likelihood of exaggeration [137]. Figure 9 shows some of the pathways by which a more encouraging global output would become possible.

Nuclear power also has some prominent advocates, including James Lovelock, Bill Gates and James Hansen ( http://www.climatechronicle.com/2010/07/james-hansen/). There are claims that a new generation of breeding nuclear reactors will provide abundant, safe power [138]. However, an assessment in 1996 undertaken by the United States National Academy of Sciences, commissioned by the US Department of Energy, concluded that breeder reactors have very high costs and marginal benefits. The Obama Administration and the USA continue to express this scepticism [139].

New communication and other technologies such as solar energy, better desalination, smart grids and batteries are useful, but insufficient to solve these growing problems. Much of the information being shared by new communication technologies is false, distracting or in other ways unhelpful. New ways of thinking and behaving are urgently needed, including as embodied in the phrase “One Health, One World^1^” ( http://www.oneworldonehealth.org/).” Indeed, the dependency on technological solutions may need to be complemented with the notion of voluntary simplicity and the need for contraction and convergence in rich and poor economies, as appropriate.

Spreading these new ideas need requires leadership and effort, and is vital if civilisation is to be sustained. A new global consciousness – a noösphere [140] - is evolving, but it needs acceleration. While systems thinking is needed, there is still room for reductionism. There appear large cognitive barriers to thinking holistically, though systems thinking may not be so alien to many health workers, trained to understand health as a system. This thinking needs broadening beyond the individual, herd, flock or population, to embrace all of life in a vast milieu, even if we are then forced to work in an essentially reductionist way.

To paraphrase René Dubos, we might have to think systemically, yet mostly operate in ways that are reductionist. But, our leaders and economists must think systemically, lest this century indeed becomes the final one with an advanced civilisation, as Lord Rees, the former president of the Royal Society has argued, may be plausible [141]. Humanity has faced crises before. Hope remains, and it is essential to maintain hope. We may yet muddle through this crisis, but only if we awaken to our otherwise inevitable peril [142].

## Endnotes

^1^This concept refers to an interdisciplinary, cross-sectoral approach to addressing human and animal health, underpinned by environmental stewardship. It is a trademark of the Wildlife Conservation Society.

## Supplementary Material

Additional file 1 Multilingual abstracts in the six official working languages of the United Nations. Click here for file
