# Supplementary material for: Infectious disease emergence and global change: thinking systemically in a shrinking world
Source: Infect Dis Poverty. 2012 Oct 25;1:5. doi: 10.1186/2049-9957-1-5 (PMC3710192; doi:10.1186/2049-9957-1-5)
Supplement: Additional file 1 — Multilingual abstracts in the six official working languages of the United Nations. [file 2049-9957-1-5-S1.pdf]

Translation of the abstract into the six official working languages of the United Nations

ظهور أمراض والتغيّر العالمي: التفكير بانتظام في عالم متقلّص

كولن د، باتلر

الخلاصة

الأساس: ثمة قلق متزايد حول ظهور أمراض معدية وتغيّرات بيئية عالمية يمكنها أن تولّد مجموعة من أوبئة بشرية خطيرة في المستقبل.

الطريقة: أجري استعراض أدبي مركز استمدّت معلوماته جزئياً من تقرير تقني قادم أعدته منظمة الصحة العالمية في موضوع البيئة والزراعة والأمراض المعدية الناجمة عن الفقر.

النتائج: هناك ما يفوق العشر فئات من ظهور أمراض معدية لكن أيّ منها لا يحلل عبء المرض الماضي أو الحاضر أو المستقبلي. كما يوحي دليل آخر أن الشاغل السائد حول الصحة العامة يركز على مجموعتين غير رسميتين. والأكثر أهمية هو التهديد المُدرك لأمراض معدية جرى التعرّف عليها حديثاً، خصوصاً فيروسات تظهر أو يتم اكتشافها حديثاً في البلدان النامية والناشئة في مخلوقات دخيلة على البلدان المتقدّمة، شأن الثدييات الغير بشرية والوطاويط والقوارض. يمكن لهذه المُسببات للمرض أن تنتقل عبر الحشرات أو الوطاويط أو من خلال الاحتكاك المباشر بلحم حيوانات الأدغال. أما الفئة الثانية فهي سلالات جديدة من الإنفلونزا الناشئة من دجاج أوخنازير التي تتم تربيتها بكثافة، أو ناشئة من الأسواق الزراعية التي تغسل بالماء وتحتك بها عن كثب عدة أنواع من الطيور.

تبدو الفئتان مبررتين نتيجة وبائين خطيرين هما فيروس نقص المناعة البشرية /الإيدز (الذي يبدو أنه نشأ من صيد حيوانات الأدغال في أفريقيا قبل الظهور عالمياً) والإنفلونزا الإسبانية التي قتلت ما يصل الى 2،5% من المجتمع الإنساني في الفترة القريبة من نهاية الحرب العالمية الأولى. لم تُمنح مساهمة البيئة الفريدة التقدير الكافي ما سهّل نمو عبء المرض في هذين الوبائين. إضافة الى ذلك، القلق الزائد حيال أمراض معدية ناشئة يصرف الانتباه عن مسائل أكثر أهمية وتتعلق بالصحة العامة، خصوصاً: (أ) الأمراض المعدية الحالية (بما فيها المهملة) و(ب) البيئة المتغيّرة التي تعرّض محددات المناعة والصحة العامة للتآكل والذي تتسبب به التغيّرات الضارة للبيئة العالمية، بما في ذلك تغيرّ المناخ وعناصر أخرى من الحياة المجهدة والأنظمة الداعمة للحضارة.

الاستنتاجات: إن التركيز على منشورة المسببات الجديدة للأمراض والأشكال الثانوية لمقاومة مضاد الميكروبات لا يبرره العبء الحالي والمحتمل للمرض ويصرف الانتباه عن مشاكل ومحددات صحية ذات أهمية أكبر. هناك فهم غير كاف لعوامل نظامية تعزز الأوبئة. قد يولّد التغير العالمي الضار ظروف مفضية الى أوبئة مستقبلية ذات عبء مرضي كبير تنشأ من خلال مقاومة مضاد الميكروبات ومبيد الحشرات، نقص التغذية، النزاع، وانهيار الصحة العامة.

Translated from English version into Arabic by Liliane Hatem, through

TRANSLATORS
WITHOUT BORDERS

疾病突现与全球变化：世界衰退背景下的系统思考

Colin D Butler

摘要

背景：对于突然出现的传染病与全球环境变化并可能导致一系列重要疾病大流行，有着日益加剧的关切。

方法：部分基于即将发表的世界卫生组织关于环境、农业和贫穷相关传染病的技术报告，进行专题文献综述。

结果：在已经出现的十多种传染病中，没有规范分析过任何一种病的疾病负担的过去、现在和未来。其它证据表明，占主导地位的公共卫生关切集中于两个非正式类群。最重要的一类是意识到新近认识的感染威胁，特别是在发展中国家出现的或新发现的病毒，对发达国家而言源于外来物种，例如非人灵长类动物、蝙蝠和啮齿类。这些病原体可能由昆虫或蝙蝠传播、或经由丛林生物直接接触传播。第二类是新型流感，由集中养殖的鸡或猪引起、或出现于与若干禽类有密切接触的亚洲菜市场。

由于两次大流行，两个分类似乎都合理：一是艾滋病毒/艾滋病，在全球性流行之前，它似乎源于非洲的丛林狩猎；一是西班牙流感，在第一次世界大战结束前后导致 2.5%的世界人口死亡。然而，在这些大流行中推高疾病负担的独特背景的作用并未得到完全认同。而且，对于突发传染病的过度焦虑转移了对重大公共卫生问题的注意力，尤其如：1）现有的（包括被忽视的）传染病，2）全球环境的恶性变化侵蚀着免疫力相关因素及公共卫生，包括气候变化、生活压力和文明支撑体系的其它方面。

结论：突发疾病的文献中对新病原体和轻型抗药性的关注并没有被其实际的和潜在的疾病负担所论证，且分散了对重要得多的卫生问题及其决定因素的注意力。对促成大流行的系统因素了解不够。不利的全球变化可能形成有助于高负担的全球疾病大流行的条件，起始于对抗菌药和杀虫剂的抗性、营养不良、局部冲突、以及公共卫生解体等。

Translated from English version into Chinese by Yi Feng-Yun, through

CHINESE CENTER FOR DISEASE CONTROL AND PREVENTION
NATIONAL INSTITUTE FOR PARASITIC DISEASES

Disease emergence and global change: thinking systemically in a shrinking world[1]

Colin D Butler

Résumé

Contexte : nous constatons une inquiétude croissante à propos des maladies infectieuses émergentes et des changements environnementaux à l'échelle mondiale capables de générer un arsenal de futures pandémies humaines.
Méthode : il a été procédé à une revue ciblée de la littérature basée en partie sur le rapport technique de l'OMS à paraître consacré à l'environnement, l'agriculture et les maladies infectieuses de la pauvreté.
Résultats : il existe plus de dix catégories d'émergence de maladies infectieuses mais aucune d'entre elles n'analyse formellement la charge de morbidité passée, présente ou à venir. Un autre indice suggère que la préoccupation en matière de santé publique se concentre plus sur deux groupes informels. La catégorie la plus importante est la menace perçue constituée par les infections récemment reconnue, notamment les virus qui surviennent ou qui ont été récemment découverts dans les pays en développement et qui proviennent d'espèces exotiques aux pays industrialisés comme les primates non humains, les chauves-souris et les rongeurs. Ces agents pathogènes peuvent être transmis par l'intermédiaire des insectes, des chauves-souris ou par contact direct avec le gibier. La seconde catégorie est constituée par les nouvelles souches de grippe surgissant en premier lieu dans les élevages intensifs de poulets, cochons ou émergeant à partir des «marchés de produits frais» asiatiques où de nombreuses espèces d'oiseaux sont en contact étroit.

Ces deux catégories paraissent justifiées en raison de deux grandes pandémies : le VIH/SIDA (qui semble avoir pris ses origines dans la chasse au gibier en Afrique avant de se répandre dans le monde entier) et la grippe espagnole qui décima jusqu'à 2.5 % de la population humaine peu après la fin de la Première Guerre Mondiale. La contribution d'un milieu unique ayant facilité la charge de morbidité élevée de ces pandémies n'a pas fait l'objet d'une appréciation suffisante. De plus, un excès d'inquiétude à propos des maladies infectieuses émergentes détourne l'attention à propos des problématiques de plus grande

[1] En français : « Émergence de maladies et changements environnementaux mondiaux : adopter une approche systémique dans un monde où les distances se réduisent ».

importance en termes de santé publique, notamment : (i) les maladies infectieuses existantes (y compris celles qui sont négligées) et (ii) le milieu en évolution qui érode les déterminants d'immunité et de santé publique causé par des changements environnementaux mondiaux négatifs, y compris le changement climatique et d'autres composants des systèmes nécessaires à la vie humaine et à la civilisation qui sont soumis à des contraintes.
Conclusions : La concentration sur les nouveaux agents pathogènes et les formes mineures de résistance aux agents antimicrobiens dans la littérature consacrée aux maladies émergentes n'est pas justifiée par leur charge de morbidité réelle et potentielle et détourne l'attention qui devrait être portée sur des problèmes et des déterminants sanitaires bien plus importants. La compréhension des facteurs systémiques qui encouragent les pandémies est insuffisante. Les changements climatiques mondiaux négatifs pourraient être à l'origine de circonstances propices à l'émergence de futures pandémies affichant une charge de morbidité élevée, survenant à cause d'une résistance aux agents antimicrobiens et aux insecticides, de cas de dénutrition, de conflits et d’une dégradation des structures de santé publique.

Translated from English version into French by Eric Ragu, through

TRANSLATORS
WITHOUT BORDERS

Всплеск болезней и глобальные изменения: мыслить систематически в сжимающейся вселенной

Колин Д. Батлер

Тезис

Предпосылки. Не может не привлечь внимания участившиеся всплески инфекционных заболеваний и глобальных климатических изменений, которые могут повлечь за собой ряд крупных эпидемий в будущем.

Методы. Помимо представленного технического доклада Международной Организации Здоровья по состоянию среды, агрикультуры и инфекционных болезней среди бедного населения планеты был проведен целенаправленный обзор литературы на данную тему.

Результаты. Существует более 10 категорий возникновения инфекционных заболеваний, но ни одна из них не характеризует их прошлые, настоящие и будущие последствия. Исследования показывают, что главная забота о здоровье населения фокусируется на двух пунктах. Во-первых, это новые зарегистрированные заболевания, в особенности вирусы, которые обнаруживаются или возникают в развивающихся странах мира, распространяемые приматами, летучими мышами и грузынами - существами, которые не так часто встречаются в развитых странах мира. Эти болезнетворные микроорганизмы переносятся летучими мышами и грузынами или вследствие прямого контакта с мясом диких животных. Вторая категория - это новые виды гриппа, возникающие среди массово разводимых свиней и цыплят, или же распространяющиеся с азиатских рынков, где разные виды домашней птицы располагаются в тесной близости друг от друга.

Выделение обеих категорий представляется оправданным, если опираться на историю двух главных эпидемий в мире: СПИД/ВИЧ (который, вероятно, произошел из мяса диких животных в Африке, а потом распространился по всему миру) и испанского гриппа, который убил около 2,5% человеческого населения под конец Первой Мировой Войны. Весьма недооценивается роль социальной обстановки в распространении этих эпидемий. Кроме того, повышенная озабоченность распространением этих болезней отвлекает внимание от других проблем поддержания общественного здоровья: в особенности: (а) от существующих (и находящихся в запущенном состоянии) инфекционных заболеваний и (б) от изменений в обществе, которые наносят ущерб основным системам охраны общественного здоровья и которые

вызваны неблагоприятными изменениями окружающей среды, включая климатические перемены и другие компоненты стрессовой жизни и технологически развивающегося общества.
Заключение: В литературе, касающейся новых возникающих заболеваний, фокус на новых болезнетворных микроорганизмах и незначительных формах резистентности микроорганизмов не представляется оправданным серьезностью этих болезней, как существующих, так и потенциальных, и отвлекает внимание от более серьезных проблем и основ общественного здоровья. Существует значительное недопонимание факторов, которые ведут к всплеску эпидемий. Неблагоприятные глобальные изменения могут породить условия, которые приведут впоследствии к эпидемиям тяжелых болезней, возникающих в результате пониженного сопротивления воздействию микробов и насекомых, недоедания, конфликтных ситуаций и снижения уровня охраны общественного здоровья.

Translated from English version into Russian by Elanorielle, through

TRANSLATORS
WITHOUT BORDERS

La aparición de enfermedades y el cambio global: pensar de forma sistemática en un cada vez más pequeño

Colin D Butler

Resumen
Contexto: Cada vez hay más preocupación por las enfermedades infecciosas emergentes y los cambios ambientales planetarios que podrían generar en el futuro una serie de pandemias humanas importantes.
Método: Se llevó a cabo una evaluación más selectiva de la literatura, en parte por el próximo Informe Técnico de la OMS sobre el medio ambiente, la agricultura y las enfermedades infecciosas de la pobreza
Resultados: Existen más de diez categorías de aparición de enfermedades emergentes, pero no se ha analizado formalmente la carga pasada, actual o futura de morbilidad. Otras pruebas sugieren que la preocupación dominante en la salud pública se concentra en dos grupos informales. Lo más importante es la percepción de que las recién descubiertas enfermedades representan una amenaza, en especial los virus que surgen o se han descubierto recientemente en los países en desarrollo, que se originan en especies exóticas en los países desarrollados, como primates no humanos, murciélagos y roedores. Estos patógenos los pueden transmitir insectos o murciélagos, o a través del contacto directo con la carne de animales salvajes. La segunda categoría son las nuevas cepas de la gripe que surgen de gallinas o cerdos criados intensamente, o que aparecen en los «mercados de aves» de Asia donde varias especies de aves mantienen un contacto estrecho.

Ambas categorías parecen estar justificadas debido a dos grandes: el VIH/SIDA (que parece que se originó de la caza de animales salvajes en África antes de su aparición a nivel mundial) y la gripe española, que mató al 2,5% de la población humana a finales de la Primera Guerra Mundial. No se tuvo en cuenta lo suficiente la contribución del entorno único que facilitó la elevada carga de morbilidad en estas pandemias. Además, el exceso de ansiedad que despiertan las enfermedades infecciosas emergentes desvía la atención de cuestiones más importantes para la salud pública, en especial: (i) enfermedades infecciosas existentes (incluyendo las olvidadas) y (ii) el entorno cambiante que está minando los determinantes de la inmunidad y salud pública, que están causados por los cambios planetarios adversos, incluyendo el cambio climático y demás componentes estresados de los sistemas vitales y de soporte a la civilización.

Conclusiones: La atención en los patógenos nuevos y las formas menores de resistencia a los antibióticos en la literatura sobre enfermedades emergente no se justifica por su carga de morbilidad, actual y potencial, y desvía la atención de los problemas y determinantes sanitarios mucho más importantes. Existe un entendimiento insuficiente de los factores sistemáticos que promueven las pandemias. El cambio planetario adverso podría generar circunstancias propicias para pandemias futuras con una alta carga de morbilidad, al surgir de la resistencia a los antibióticos e insecticidas, la desnutrición, los conflictos y el desmoronamiento de la salud pública.

Translated from English version into Spanish by Lizette Britz, through

TRANSLATORS
WITHOUT BORDERS
